# Time-Frequency Analysis of Mu Rhythm Activity during Picture and Video Action Naming Tasks

**DOI:** 10.3390/brainsci7090114

**Published:** 2017-09-06

**Authors:** Megan E. Cuellar, Christina M. del Toro

**Affiliations:** Department of Speech-Language Pathology, Midwestern University, 555 31st Street, Downers Grove, IL 60304, USA; cdeltoro@midwestern.edu

**Keywords:** action naming, verbs, EEG, mu rhythm, event related spectral, perturbations, sensorimotor integration

## Abstract

This study used whole-head 64 channel electroencephalography to measure changes in sensorimotor activity—as indexed by the mu rhythm—in neurologically-healthy adults, during subvocal confrontation naming tasks. Independent component analyses revealed sensorimotor mu component clusters in the right and left hemispheres. Event related spectral perturbation analyses indicated significantly stronger patterns of mu rhythm activity (*p*FDR < 0.05) during the video condition as compared to the picture condition, specifically in the left hemisphere. Mu activity is hypothesized to reflect typical patterns of sensorimotor activation during action verb naming tasks. These results support further investigation into sensorimotor cortical activity during action verb naming in clinical populations.

## 1. Introduction

Studies show that action observation and action execution elicit similar patterns of cortical activity in sensory and motor regions of the brain [1,2,3]. Mirror neurons, first discovered in the F5 area of macaque monkeys [3]—and shortly thereafter in humans—in Broca’s Area, the inferior frontal gyrus (IFG), and in the inferior parietal lobule (IPL), are considered the “neural mechanism” that links observation with execution, and drives imitative motor learning processes [1,4,5,6,7]. Specifically, mirror neuron activity occurs in response to a variety of perceptual conditions including, but not limited to, viewing pictures and videos of hand, leg, and foot actions [4,8,9], emotional facial expressions [10,11], oropharyngeal swallowing movements [12,13], and orofacial speech movements [14]. Recently, additional cortical areas such as the dorsal premotor cortex (PMC), superior parietal lobule (SPL), medial temporal gyrus (MTG), superior temporal sulcus (STS), anterior cingulate cortex (ACC), and the insula, have also been shown to activate during action observation-execution experiments (see Rizzolatti & Craighero [1] for a detailed review). Evidence of the so-called “extended mirror neuron network” has led to more recent notions that mirror neuron activity goes beyond motor learning by associating sensorimotor representations of actions with corresponding cognitive-linguistic representations of actions (i.e., action names).

### 1.1. Mirror Activity in Response to Action Verbs

Evidence for mirror neuron activity during linguistic processing of action verbs has steadily increased in the past decade. Studies have shown that simply reading an action verb such as kick or lick, activated areas of the premotor cortex that control the execution of leg and face movements [15,16]. A seminal study conducted by Pulvermuller, Harle, and Hummel [17], provided evidence for the localization of mirror neuron activity within the primary motor cortex, which corresponded with the particular body-part(s) that perform the action. Specifically, during a lexical decision task, leg-related verbs activated the vertex of the motor strip, while face-related verbs activated over the left Sylvian fissure near the representation of the articulators [17]. Overlap between the neural substrates responsible for action observation, action execution, and the linguistic processing of action verbs was further demonstrated in a study by Andric and colleagues [18] that used magnetic resonance imaging (MRI) to compare the location and strength of mirror neuron activity during the observation of symbolic emblems (e.g., thumbs up), grasping actions (e.g., holding a cup), and speech (e.g., “it’s good”). The authors found activity in the STS and MTG, in addition to the classical frontal and parietal mirror neuron areas, thereby demonstrating shared sensorimotor and linguistic neural substrates for gestures and speech production [18]. Another neuroimaging study provides evidence that handedness factors into the hemispheric localization of mirror neuron activity. A functional magnetic resonance (fMRI) study of left- and right-handed neurologically healthy adults revealed that mirror neuron activity in the premotor cortex was stronger in the hemisphere contralateral to participants’ dominant hands [19]. This pattern was seen when participants made lexical decisions regarding the name of manual-action words, as well as when participants imagined themselves performing the actions. Such findings lead the authors to conclude that the semantic aspects of manual-action verb processing may vary in accordance with the way in which an individual typically performs actions, further supporting an interaction between the sensorimotor representation (mirror neurons) of actions and the linguistic (semantic) representation of action verbs in an individualized manner.

### 1.2. Stimuli Effects

Despite the number of studies of that have documented mirror neuron activity in response to viewing, imagining, performing, and linguistically processing actions, it must be acknowledged that the quality and mode of stimuli delivery yields contrasting results in terms of the localization, strength, and timing of activity. For instance, weaker patterns of mirror neuron activity have been found in response to viewing pictures when compared to viewing videos of hand actions [20]. However, different modes of video display have elicited similar patterns of mirror neuron activity including, but not limited to, animated physiology videos [21], point light biological action videos [22], cartoon videos of action execution [23], and videos of humans performing actions [24,25]. Pictures or videos depicting non-goal related actions (e.g., hand extension) [26], biologically irrelevant actions (e.g., barking) [27], or incomplete actions whereby the action does not reach the desired end-point [28] have been shown to elicit significantly weaker patterns of mirror neuron activity. Such evidence seems to indicate that mirror neuron activity varies under different conditions, although in general the more realistic the stimulus—i.e., actions that are biologically relevant, complete, presented in videos—the stronger the response.

Just as the nature of the action stimuli may influence the strength of mirror neuron activity, the nature of the linguistic stimuli may also reveal differences in the strength or location of mirror neuron activity. For example, transitive verbs elicit stronger patterns of mirror neuron activity than intransitive verbs, particularly in the posterior regions of the parietal lobe and in the left inferior frontal gyrus [29]. Consistent with previous literature comparing videos and pictures, the difference between transitive and intransitive verbs was even greater when the actions were presented in video segments as compared to when actions were represented in line drawings. Specifically, greater activation was seen in the right inferior and superior parietal cortices when the action verbs were observed in videos [29]. These results suggest mirror neuron activity is specifically related to actions performed on an object, and thus will not necessarily be activated during the processing of all verbs.

The study by den Ouden and colleagues [29] is noteworthy for a methodological change compared with previous investigations of action observation. The participants in previous studies performed comprehension tasks such as reading or lexical decision, while the participants of the den Ouden et al. [29] study verbally named the action presented in picture and video. Differences in the activation patterns seen in fMRI during picture and video presentation suggest videos are a more natural representation of actions and require less processing, while pictures required more visual scanning and strategy due to the lack of movement. Furthermore, video observation led to activation of Wernicke’s area, which was not activated during picture observation. Thus, linguistic processing areas were engaged during the more natural video presentation of actions. Furthermore, participants verbally named the actions. Consequently, the den Ouden et al. [29] study extends previous literature on mirror neuron activity during comprehension tasks to verbal tasks. However, more research is needed to understand the role of mirror neuron activity (i.e., sensorimotor activity) in word retrieval—specifically, identifying when sensorimotor processes are active in the time course of action verb naming.

### 1.3. Neurophysiological Measures

Mirror neuron activity has been investigated using a variety of neuroimaging techniques such as magnetic resonance imaging (MRI), functional magnetic resonance imaging (fMRI), electroencephalography (EEG), and magnetoencephalography (MEG) to determine the location and strength of the activity of sensorimotor processing in a variety of action-observation, -execution, and verb processing tasks. An additional measure of interest, particularly in relation to verb processing, relates to the temporal dynamics of mirror neuron activity. In 2004, Hauk and Pulvermuller [30] measured the time-course of neural activity during passive reading of actions performed via face, arm, and leg movements by analyzing event-related potentials (ERPs) using EEG. The passive reading of action verbs resulted in somatotopically organized activation of areas within the motor cortex approximately 200 ms after stimulus onset. This timing of the mirror neuron activity during verb reading matches the proposed word processing model by Indefrey and Levelt [31] in which lexical-semantic access occurs within 200 ms of stimulus presentation. A meta-analysis conducted by Indefrey and Levelt [31], which included 82 experiments, revealed consistent findings for the spatial and temporal aspects of word production. Using the reaction time and ERP data as predictors, the authors analyzed the findings of MEG studies to determine if the predicted time course of word production aligned with the spatial components of word production. The results of the meta-analysis did support the hypothesized time course of lexical access at 175–250 ms; phonological retrieval 250–330 ms; and syllabification 330–445 ms. Thus, the results presented by Hauk and Pulvermuller [30] provide evidence that the action semantics of the action words were accessed during lexical-semantic processing. Given the assumption that action semantics are supported by mirror neuron activity as the mechanism for the sensorimotor representation of actions, these findings are further evidence to support a link between the sensorimotor representation and the linguistic representation of actions. Furthermore, the identification of the time course of mirror neuron activity during action verb processing suggests the sensorimotor representation of actions is linked to, or even a component of, the semantic representation of action verbs. However, ERP studies do not depict changes in neural activity across the time course of lexical processing. Therefore, a more specific means of analyzing the time course of mirror neuron activity during naming tasks may elucidate sensorimotor mechanisms that are thought to support action semantics.

### 1.4. Mu Rhythm Activity

A number of electrophysiological studies using whole head EEG and MEG imaging techniques have shown that mu rhythm suppression provides a valid measure of mirror neuron activity. The combined “mu rhythm” is characterized by an initial spectral peak in the ~10 Hz “alpha” frequency band and a second spectral peak in the ~20 Hz “beta” frequency band, and is considered a global measure of cortical sensorimotor activity [32,33,34,35,36,37]. As with other rhythms, mu rhythm activity is measured via time-locked decreases and increases in the signal amplitude reflecting desynchronization (i.e., neural activity) or synchronization (i.e., neural inhibition or neural idling) respectively [38,39].

Independent component analysis (ICA) has been shown to separate and sort EEG signals into temporally independent and spatially fixed components [40]. However, one of the limitations of EEG signal analysis is the presence of movement artifacts due to eye blink and gross motor movements [41,42]. Recently, ICA has been shown to effectively identify and remove movement artifacts from neural signal data by using higher-order statistics, such as kurtosis [43,44].

A number of studies show that ICA can be used to identify sensorimotor activity via localized mu components. Clusters of mu components have been identified and localized during the perception or imagination of movement in premotor and/or primary sensorimotor cortices [45,46,47,48,49]. In addition to using ICA to spatially map mu rhythm activity, patterns of mu ERS/ERD may be analyzed using event-related spectral perturbations (ERSPs). ERSPs provide a means to visually analyze patterns of ERS/ERD in frequency bands across time, relative to the onset of a particular time-locked event [39,40]. Generating a color-coded time-frequency graphic, ERSPs depicting the average changes in spectral power between the baseline time period and the experimental condition time period are plotted across subjects and conditions [50,51]. Hence, ERSPs can be used to compare and contrast dynamic changes in mirror neuron activity, exhibited by clusters of mu rhythm independent components that exhibit spectral peaks at ~10 Hz (mu-alpha) and ~20 Hz (mu-beta) respectively.

Recent studies have successfully identified mu rhythm activation during processing of action semantics whether presented visually, auditorily, or orthographically. In 2013, Moreno, Vega, & Leon [52] found that action language modulated mu rhythm suppression similarly to action observation. Participants performed a recognition task with action sentences, abstract sentences, and action videos, in which they were asked to press a button if the sentence or video had been previously seen. Results showed greater mu suppression during the action video and action sentence conditions compared to the abstract sentence condition. Furthermore, there was no significant difference between action video and action sentence suggesting that the comprehension of action words activates the same motor areas as action observations. In a follow-up study, time frequency analyses were used to determine differences in the time course of mu suppression when participants read action sentences, abstract sentences, and perceptive (sensory) sentences. Consistent with the previous study, mu suppression was observed only during the processing of action sentences [53]. Furthermore, patterns of mu rhythm have been found during action word reading tasks in bilingual individuals with no differences between languages. Vukovic & Shtyrov [54] compared patterns of mu ERD during passive reading of action words by German-English bilinguals. While mu ERD was observed in both languages, significantly stronger patterns of mu ERD were observed when reading in the primary language. The authors suggest a stronger engagement between motor and language systems in the primary language because the action semantics were first learned through that language [54]. Together these studies support the use of mu rhythm activity to measure sensorimotor processing during action language tasks, and the notion that action language is embodied.

To date, the strongest evidence for mirror neuron activity during action verb processing involves observation of biologically-relevant actions presented in video form with names that are transitive verbs. However, previous studies have not used ERSPs to measure mu rhythm activity in these conditions, nor during naming tasks. More specific comparisons of the strength of mu rhythm activity (i.e., sensorimotor activity) across time during picture and video verb naming will further illustrate how different stimuli presentations of actions may affect the timing of word production processing. If mirror neuron activity occurs before lexical-semantic processing is theorized to occur (200 ms), then sensorimotor representations of actions may support action comprehension; however, if mirror neuron activity occurs at the same time as linguistic processing, then sensorimotor representations may be hypothesized to support lexical access, providing evidence of a direct link between sensorimotor representations and lexical-semantic representations of actions. Such a link would have implications for the assessment and treatment of action verb impairments in acquired communication disorders, such as aphasia. In order to investigate differences in the neurophysiological response to action verbs presented in video and picture forms, this study used whole-head electroencephalography (EEG) to measure changes in the amplitude and timing of mu rhythm activity in neurologically-healthy adults during a confrontation naming task of actions depicted in videos and pictures. Specifically, this study aims to (1) use ICA to identify bilateral mu components during subvocal naming of action pictures and videos, and (2) use ERSPs to compare and contrast the strength and timing of sensorimotor activity during subvocal naming of action pictures and videos.

## 2. Methods

### 2.1. Participants

A repeated measures design was used to analyze patterns of mu rhythm ERS/ERD obtained from 21 neurologically healthy adults during the sub vocal naming of actions depicted in videos and pictures. The experimental protocol was approved by the Midwestern University IRB board, and all participants gave their informed consent prior to participating in the study. Participants had no self-reported history of developmental or acquired cognitive or communication impairments. Additionally, each participant completed a demographic questionnaire to provide information regarding handedness, language, age, and gender. Table 1 presents the participant demographics. The short form of the Boston Naming Test (BNT) [55] and the Montreal Cognitive Assessment (MOCA) [56] were also administered to screen for cognitive-linguistic deficits that may interfere with performing the action verb naming task. All participants scored as unimpaired on the BNT and MOCA (see Table 1 for average scores).

### 2.2. Stimuli

**Figure 1:**
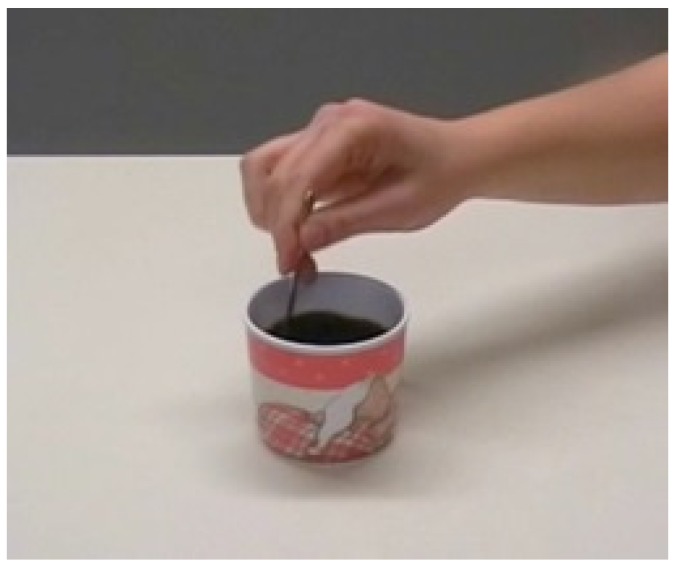
The “stirring” picture stimulus is displayed which was taken as a screenshot from the original stirring stimulus video.

Videos were taken from an online database of hand-related actions [57] and edited using iMovie [58] to create a 2-s stimulus representing each action. Subsequently, screenshots of the videos were taken to create corresponding pictures for each action (see Figure 1 for an example). Of the 1074 video clips of actions that were available in the online database created by Umla-Runge and colleagues [57], 22 biologically-relevant action verb video clips were initially selected as stimuli for this study based on three criteria—recognizable action in picture and video format, name agreement, and similar psycholinguistic criteria of concreteness, familiarity, imageability, and frequency. First, the researchers chose actions which could be identified from a still shot of the video using three independent reviewers. The reviewers identified still shots which could easily be recognized as the action performed in the video. Actions which were agreed upon by all reviewers were selected. Name agreement data was collected from a separate normative sample of 20 neurologically healthy adults that were asked to view the action pictures and videos, and report all possible names for the actions depicted. Action videos and pictures that did not yield 100% name agreement were excluded from the study. Lastly, the actions which met criteria for recognition in picture and videos and name agreement were analyzed for psycholinguistic values. Because the number of stimuli were limited, the aim was to include actions with relatively similar psycholinguistic values using averages and standard deviations. Values for concreteness, frequency, familiarity and imageability were initially taken from the MRC Psycholiguingstic Database [59] which reports Francis and Kucera [60] values for frequency and concreteness. The updated frequency and concreteness values by Brysbaert and New [61] and Brysbaert, Warriner, and Kupperman [62] were subsequently collected. In all, 20 action pictures and videos were retained as experimental stimuli. The final 20 action verbs and the values for the psycholinguistic criteria are presented in the Appendix A (Table A1). All actions included were transitive verbs, based on results from previous research suggesting actions upon objects evoke a stronger mirror neuron response [29]. All stimuli were formatted for presentation on a 21.5 inch HD Dell computer screen located approximately 3 feet in front of the participant.

### 2.3. Data Collection

EEG data was collected in a double walled, soundproof audio booth using E-Prime 2.0 software [63] to present and time-lock action picture and video stimuli during the continuous recording of EEG data. Event-related EEG data was collected using 64 electrodes via a 64 channel HydroCell geodesic sensor net (HCGSN), arranged in accord with the international 10–20 system. EEG channel data was recorded using EGI Netstation 4.5.7 software (Electrical Geodesics, Inc., Eugene, OR, USA). The following time-locked events were marked during each trial: (a) 2000 ms inter-trial interval with blank screen, (b) 1000 ms fixation cross, (c) 2000 ms picture or video presentation of an action that participants were asked to silently name in their head, (d) 100 ms fixation cross, (e) 2000 ms picture or video presentation of an action that participants were asked to name aloud, and (f) 1000 ms presentation of a stop sign. Thus, participants were asked to subvocally name the first presentation of the action picture or video in order to minimize any potential noise associated with movement artifacts. Subsequently, participants were asked to name aloud the second presentation of the same action picture or video so as to encourage consistency regarding action naming across all trials.

Videos and pictures were randomly presented in separate blocks and the order of stimuli condition blocks, pictures vs. videos, were also randomized for each participant. Each block presented all 20 verbs, and each block was repeated three times resulting in 60 trials per participant, per stimulus condition. A break was offered to participants after each block to prevent fatigue.

### 2.4. Data Analysis

Using EEGLAB, an open source Matlab toolbox [64], raw EEG data were down sampled at 256 Hz, and channels were registered using BESA head model coordinates. Data were re-referenced to the average common reference, and were band passed filtered between 7 and 30 Hz. The data were epoched to begin at 1000 ms prior to the time-locked the presentation of pictures or videos that demarcated the onset of subvocal naming (i.e., time point zero), and ended at 2000 ms, yielding a baseline of 1000 ms and a total sum of 3000 ms of experimental data in each trial. Epochs were subsequently visually inspected and those that were contaminated with noise due to movement were rejected from the dataset. A minimum of 40 epochs were retained per condition for each participant.

Following preprocessing, each condition dataset was concatenated and underwent ICA [65] using the extended version of the “runica” algorithm in the EEGLAB v13.4 (SCCN, San Diego, USA) ICA toolbox. Each participant yielded 64 independent components (ICs) per condition [50]. After “unmixing” signal data to obtain independent components (ICs), which are considered spatially fixed plots of component activity projected onto topographic scalp maps [64], group analyses were performed using the STUDY toolbox in EEGLAB v13.4.

For study group analyses, component measures were precomputed based upon the spectral characteristics of IC activity and the scalp map spatial distribution of each IC [65]. A “pre-clustering array” was created to instruct the principle component analyses (PCA) to cluster ICs demonstrating similar spectral and scalp map characteristics across participants [64]. Independent components that exhibited incongruent topographic and spectral characteristics were excluded from clusters. Following IC clustering, each IC that was included in right (R) and left (L) hemisphere mu clusters, were visually inspected to ensure that each IC met the following criteria: (1) topographic scalp maps exhibited a localized pattern of signal activity in the appropriate (R vs. L) hemisphere, and (2) power spectra displayed peaks in the alpha (~10 Hz) and beta (~20 Hz) frequency ranges. In addition, other component clusters were individually inspected to ensure that all mu components were appropriately assigned during PCA clustering.

To analyze the power of ERS/ERD in the alpha (8–13 Hz) and beta (15–25 Hz), or the combined mu rhythm exhibited by the ICs that were included in the R and L mu clusters across time, ERSPs were obtained within the 7–30 Hz frequency range. ERSP analyses were conducted. The time-frequency analyses were analyzed using a Morlet sinusoidal wavelet transformation, set at 3 cyles and linearly increasing to 20 cylces at 30 Hz [57]. Dynamic changes in the power of alpha and beta frequency ERS/ERD, occurring 1000 ms prior to the time-locked event (i.e., picture and video subvocal naming) and continuing up to 2000 ms following the time-locked event, were of particular interest (i.e., beginning 1000 ms prior to the onset of picture/video subvocal naming and continuing throughout the duration of the epoched trials). ERSPs were computed relative to the baseline, which was computed from 200 randomly sampled latency windows from each inter-trial interval. To statistically analyze conditional effects between (1) subvocal naming of action pictures, and (2) subvocal naming of action videos, EEGLAB bootstrapping statistics were employed with an alpha value set at 0.05 [66,67]. False discovery corrections (FDR) were applied to adjust for multiple hypotheses [68].

## 3. Results

**Figure 2:**
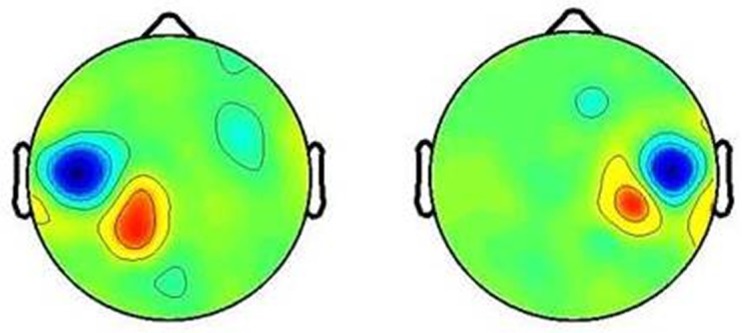
Left and right mu component cluster topographic scalp maps.

**Figure 3:**
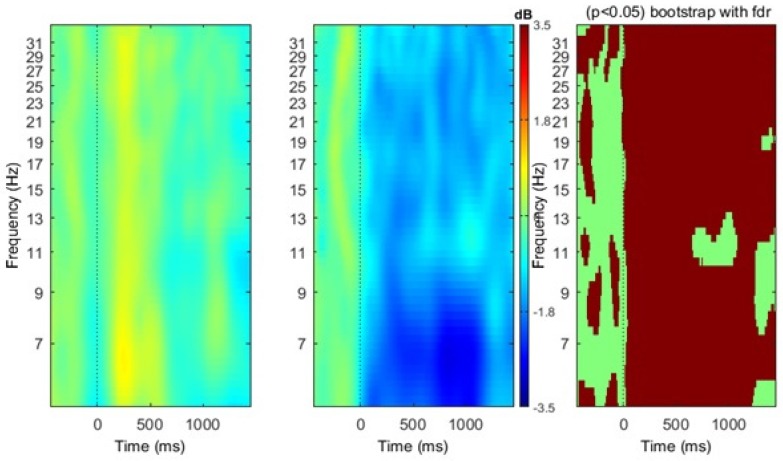
Left mu cluster event-related spectral perturbations (ERSP) results. The first and second panels depict ERD, indicated by blue coloring, and ERS, indicated by red coloring, across time and frequency for picture and video verb naming conditions, respectively. The third panel depicts significant difference between the conditions, indicated by red coloring, (*p*FDR < 0.05) with Bonferroni corrections for false discovery rates.

**Figure 4:**
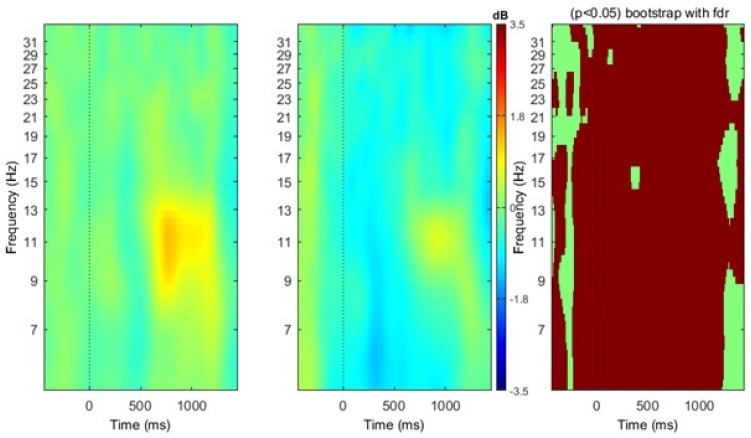
Right mu cluster ERSP results. The first and second panels depict ERD, indicated by blue coloring, and ERS, indicated by red coloring, across time and frequency for picture and video verb naming conditions, respectively. The third panel depicts significant difference between the conditions, indicated by red coloring, (*p*FDR < 0.05) with Bonferroni corrections for false discovery rates.

As hypothesized, mu component clusters were identified bilaterally (see Figure 2), as indicated by spectra peaks at ~10 Hz and ~20 Hz, respectively. ERSP analysis of both the right and left mu clusters provide evidence of mu ERD during the action picture and video subvocal naming tasks (see Figure 3 and Figure 4). Due to the statistical limitations of performing independent component analysis, activity in the right vs. left hemisphere could not be directly compared across participants. However, visual analysis of ERSP data indicate differences in the power of mu activity, with the left mu cluster clearly exhibiting more robust patterns of ERD in the alpha (~8–13 Hz) and beta (~15–25 Hz) frequency ranges. While the right cluster does not exhibit powerful patterns of mu ERD in the picture or video conditions, there were significant differences in the strength of activity (*p*FDR < 0.05) across the alpha and beta frequency ranges. In contrast, the left mu cluster exhibits significantly stronger patterns of mu ERD (*p*FDR < 0.05) in the video condition as compared to the picture condition, indicated by the increased number of time-frequency voxels that appear subsequent to time point zero (see Figure 3 and Figure 4).

## 4. Discussion

### 4.1. Localization of Mu Rhythm ERD during Action Verb Naming

The primary aim of the current study was to use a novel means of analyzing the spatiotemporal dynamics of sensorimotor activity during the subvocal naming of static (i.e., picture) vs. dynamic (i.e., video) actions. A number of studies provide evidence of mu rhythm activity [52,53,54] during action verb processing tasks. As hypothesized—and in accordance with previous studies—the current study revealed bilateral mu component clusters during action word processing and specifically in subvocal naming conditions. These results are in line with previous EEG studies in which mu suppression was found to be greater during action language processing conditions [52,53,54]. To our knowledge, there is only one previous neurophysiological study of action picture versus action video naming. In an fMRI study of action naming by den Ouden and colleagues [29], stronger patterns of motor activity were elicited during video presentation compared to picture presentation. Additionally, the video condition elicited activity in Wernicke’s area, suggesting linguistic processing is facilitated by video presentation of actions but not by picture presentation.

In the current study, minimal differences were observed regarding the time course of mu activity across conditions. However, significant differences were revealed in terms of the power or “strength” of activity. Specifically, dynamic action videos elicited significantly stronger patterns of mu activity, beginning at 0 ms and continuing throughout the duration of the naming task, which ended at 2000 ms. Considering the proposed functional link between action observation, action execution, and the lexical-semantic processing of actions [1], it seems reasonable to suggest that cortical sensorimotor activity provides a common framework for the multimodal processing of actions, which is modulated via mu rhythm activity. Furthermore, finding significant differences in the strength of mu activity in both the right and left hemispheres across conditions suggests that dynamic action videos may represent the most effective means to elicit cortical sensorimotor activity during action naming tasks. The theoretical and clinical implications of these findings will be the focus of the remaining discussion; more specifically, the timing of the interaction between action observation and action semantics within the word production process and the resulting clinical implications for treatment of word production impairments will be discussed.

### 4.2. Action Observation and Action Semantics

Across participants, the onset of sensorimotor activity during the subvocal naming tasks occurred at 200 ms, which, according to the timing theory put forth by Indefrey and Levelt [31], falls within the proposed time period of lexical-semantic access. Similarly, Hauk and Pulvermuller [30] reported sensorimotor activation at 200 ms after presentation of an action word. Pulvermuller [69] concluded that the sensorimotor activation was occurring as part of the lexical semantic processing and not post-lexically. The results of the current study further support the hypothesis that sensorimotor representations of action observation and action execution are in fact activated during the linguistic processing of action verbs, suggesting that lexical semantic access for action verbs does not solely involve linguistic representations, but also invokes supportive sensorimotor representations.

Several studies have sought to use sensorimotor activation to enhance naming impairments through the use of gestures. These studies found improvements in naming through the training of gestures related to words (i.e., a gesture for drinking or cutting with scissors), although only when combined with verbal production of the word (see Rose, Raymer, Lanyon, & Attard, [70] for a review). Other studies have reported improvement in naming when participants completed a meaningless gesture with the impaired or unimpaired arm, or when standing as compared to sitting (see Marangolo & Caltagirone, [71] for a review). However, these studies did not solely focus on action naming and resulted in limited generalization. If processing of an action verb involves an interaction between the linguistic and the sensorimotor representation of that verb, treatments that invoke the supportive sensorimotor processes could improve word production. The results of this study suggest that videos elicit stronger patterns of sensorimotor activity during subvocal action naming as compared to pictures. Therefore, incorporating video stimuli during action verb naming tasks may provide enhanced access when the lexical-semantic system is damaged, further enhancing treatment effectiveness.

Several recent investigations of the effects of action observation on word production in persons with aphasia have reported behavioral effects. The first of these experiments by Marangolo, Cipollari, Fiori, Razzano, & Caltagirone [72] compared the effects of intense verb retrieval training when participants either (1) observed actions, (2) observed and executed the same actions, or (3) observed actions and executed a meaningless movement. In all three conditions, the therapist produced the action and the participant produced the verb. Four of six participants with aphasia significantly improved verb production following only the action observation as well as action observation and execution conditions. Participants did not improve on verb production when they observed a meaningless gesture, which implies the link between actions and verbs is at the semantic level of verb processing. In a follow-up study by Marangolo and colleagues [73], persons with aphasia observed human and non-human actions and produced the corresponding verb. Participants improved with maintenance effects in naming the human actions only. This finding suggests that only biologically relevant actions have sensorimotor representations.

A study by Bonifazi et al. [74] replicated the stimulus conditions used by Marangolo et al. [73] but also included a novel condition in which the actions were presented in video-clips rather than by a live model. Notably, improvement did not significantly differ when the action was observed from a video clip. Another intriguing result from the Bonifazi et al. [74] study is that only the individuals with lexical-phonological impairments improved while the individuals with semantically-based verb impairments did not. To explain this finding, the authors hypothesized that the semantic deficit hindered the activation of the sensory-motor features of the verbs and thus verb production did not improve. The difference in treatment outcomes from the semantic impairment group is further evidence of the hypothesis that sensorimotor representation of actions interacts with the semantic representation of actions. However, a study by Faroqi-Shah and Graham [75] reported that one of two participants improved in action naming from video stimuli and suggested the difference was related to phonological impairments of the second participant, which were not directly addressed by the treatment procedures (i.e., no phonological cueing). However, the authors also posited a lower premorbid education may have led to reduced treatment effects [75]. As such, the effectiveness of video verb training for types of aphasia warrants further examination.

In summary, there is growing evidence of the interaction between sensorimotor and linguistic representations of action verbs. Furthermore, treatments that evoke this interaction consistently report improvement in action naming. It has been understood for some time that noun and verb impairments are distinct and thus, the treatment of these linguistic units should be distinct [76]. Incorporating the sensorimotor representation of action verbs in treatment stimuli is one method of differentially treating action verbs.

### 4.3. Limitations and Future Directions

To our knowledge, this is the first study to use whole-head EEG analysis techniques to map cortical sensorimotor activity via mu rhythm ERS/ERD during subvocal action verb naming tasks. However, it must be acknowledged that there are inherent differences between covert and overt naming tasks, which limit the generalization of study findings to therapeutic naming tasks. Covert naming tasks were utilized in the current study as a means to dissociate the cortical sensorimotor activity that occurs during motor speech planning and production from the cortical sensorimotor activity that is thought to facilitate lexical processing during verb naming. Thus, the current method does not directly indicate sensorimotor activity during verbal production but during silent naming. However, future studies employing surface electromyography (sEMG) in conjunction with whole-head EEG would allow researchers to simultaneously record and analyze peripheral orofacial muscle activity and cortical sensorimotor activity during overt naming tasks. Finally, a recent study reported increases in cortical language and sensorimotor activity of older adults during naming tasks, which suggests older individuals require additional processing [77]. Given that aphasia typically affects older individuals, the next logical step in this line of research is to use the current methodology to map cortical sensorimotor activity in neurologically-healthy older adults. Thus, in addition to behavioral measures of increased word production, mu rhythm activity may provide a meaningful measure of improved connections between the sensorimotor and linguistic representations of action verbs during overt naming tasks. Lastly, the findings that action observation—either with or without a model of action execution—has been shown to improve action verb naming in individuals with aphasia [72,73,74], and offers a novel area of exploration aimed at improving action verb naming treatment methodologies. Furthermore, while the studies reviewed here reported positive effects of action observation when presented live or by video, no comparison has been made between pictures and videos. Traditional naming therapy involves picture stimuli, hence, a direct comparison between video and picture action observation treatment is planned.

## 5. Conclusions

The results of the current study are consistent with previous action observation and naming literature, which has reported that videos elicit the strongest pattern of sensorimotor activity. These results further support claims of a link between the sensorimotor representation of actions and the related linguistic representation. Therefore, action naming treatments may be strengthened by incorporating videos in order to elicit the strongest pattern of sensorimotor activity.

## Figures and Tables

**Table 1:** Participant Demographics.

	Age	Education	BNT	MOCA	Handedness	Monolingual
Average	36.1	17.4	15	28.8	19 Right	17 Yes
Standard Deviation	16.1	1.6	1	1.2	2 Left	4 No

## Appendix A

**Table A1:** Action Verbs and Psycholinguistic Values.

Target	FK Conc.	BN Conc.	Familiarity	Imageability	FK Freq.	BN Freq.
Typing	376		567	395	3.24	
Pouring	356	3.8	545	495	5.92	302
Stapling		3.61			0.18	9
Cutting	430	4.11	581	460	22.08	1126
Folding		3.89			2.08	106
Throwing	400	3.69	548	477	29.18	1488
Painting	577	4.59	551	567	29.20	1489
Stirring		2.93			2.61	133
Catching		3.42			12.71	648
Hammering	605	3.85	515	618	1.57	80
Ironing	584	4.1	555	561	1.47	75
Mixing					3.45	
Mowing		3.63			0.94	48
Rolling	457	4.04	547	496	21.55	1099
Shaving		4.63			5.84	298
Stacking					0.90	
Stamping		3.66			0.55	28
Opening	381	3.79	581	425	38.43	1960
Peeling	432	3.82	507	436	1.10	56
Knitting	583	4.14	501	578	2.25	115

Values for familiarity and imageability were taken from the MRC Psycholinguistic Database [59]. Two sources of values for frequency and concreteness are displayed. FK Freq. = frequency values, FK Conc. = concreteness values both from Franics & Kucera [60] as supplied by the MRC Psycholinguistic Database. BN Freq. = frequency values taken from the database by Brysbaert, & New [61]. BN Conc. = concreteness values taken from the database by Brysbaert, Warriner, & Kuperman [62].

## References

[1] Rizzolatti G., Craighero L. (2004). The mirror-neuron system. Ann. Rev. Neurosci..

[2] Rizzolatti G., Fabbri-Destro M. (2008). Mirror neurons and mirror systems in monkeys and humans. Am. Physiol. Soc..

[3] Rizzolatti G., Fadiga L., Gallese V., Fogassi L. (1996). Premotor cortex and the recognition of motor actions. Cogn. Brain Res..

[4] Buccino G., Binkofski F., Fink G.R., Fadiga L., Gallese V., Seitz R.J., Zilles K., Rizzolatti G., Freund H.J. (2001). Action observation activates premotor and parietal areas in a somatotopic manner: An fMRI study. Eur. J. Neurosci..

[5] Chong T.T.J., Cunnington R., Williams M.A., Kanwisher N., Mattingley J.B. (2008). fMRI adaptation reveals mirror neurons in human inferior parietal cortex. Curr. Biol..

[6] Molengerphs P., Cunnington R., Mattingley J.B. (2012). Brain regions with mirror properties: A meta-analysis of 125 human fMRI studies. Neurosci. Biobehav. Rev..

[7] Rozzi S., Ferrari P.F., Bonini L., Rizzolatti G., Fogassi L. (2008). Functional organization of inferior parietal lobule convexity in the macaque monkey: Electrophysiological characterization of motor, sensory and mirror responses and their correlation with cytoarchitectonic areas. Eur. J. Neurosci..

[8] Fadiga L., Fogassi L., Pavesi G., Rizzolatti G. (1995). Motor facilitation during action observation: A magnetic stimulation study. J. Neurophysiol..

[9] Hari R., Forss N., Avikainen S., Kirveskari E., Salenius S., Rizzolatti G. (1998). Activation of human primary motor cortex during action observation: A neuromagnetic study. Proc. Natl. Acad. Sci. USA.

[10] Enticott P.G., Johnston P.J., Herring S.E., Hoy K.E., Fitzgerald P.B. (2008). Mirror neuron activation is associated with facial emotion processing. Neuropsychologia.

[11] Moore A., Gorodnitsky I., Pineda J. (2012). EEG mu component responses to viewing emotional faces. Behav. Brain Res..

[12] Ogura M., Watanabe Y., Sanjo Y., Edahiro A., Sato K., Katakura A. (2014). Mirror neurons activated during swallowing and finger movements: An fMRI study. J. Oral Maxillofac. Surg. Med. Pathol..

[13] Ushioda T., Watanabe Y., Sanjo Y., Yamane G.Y., Abe S., Tsuji Y., Ishiyama A. (2012). Visual and auditory stimuli associated with swallowing activate mirror neurons: A magnetoencephalography study. Dysphagia.

[14] Murakami T., Restle J., Ziemann U. (2011). Observation-execution matching and action inhibition in human primary motor cortex during viewing of speech-related lip movements or listening to speech. Neuropsychologia.

[15] Boulenger V., Hauk O., Pulvermuller F. (2009). Grasping ideas with the motor system: Semantic somatotopy in idiom comprehension. Cereb. Cortex.

[16] Aziz-Zadeh L., Wilson S.M., Rizzolatti G., Iacoboni M. (2006). Congruent embodied representations for visually presented actions and linguistic phrases describing actions. Curr. Biol..

[17] Pulvermuller F., Harle M., Hummel F. (2001). Walking or talking? Behavioral and neurophysiological correlates of action verb processing. Brain Lang..

[18] Andric M., Solodkin A., Buccino G., Goldin-Meadow S., Rizzolatti G., Small S.L. (2013). Brain function overlaps when people observe emblems, speech, and grasping. Neuropsychologia.

[19] Willems R.M., Hagoort P., Casasanto D. (2010). Body-specific representations of action verbs neural evidence from right- and left-handers. Psychol. Sci..

[20] Perry A., Bentin S. (2009). Mirror activity in the human brain while observing hand movements: A comparison between EEG desynchronization in the μ-range and previous fMRI results. Brain Res..

[21] Marcus N., Cleary B., Wong A., Ayres P. (2013). Should hand actions be observed when learning hand motor skills from instructional animations?. Comput. Hum. Behav..

[22] Ulloa E.R., Pineda J.A. (2007). Recognition of point-light biological motion: Mu rhythms and mirror neuron activity. Behav. Brain Res..

[23] Van Overwalle F., Baetens K. (2009). Understanding others’ actions and goals by mirror and mentalizing systems: A meta-analysis. Neuroimage.

[24] Gangitano M., Mottaghy F.M., Pascual-Leone A. (2001). Phase-specific modulation of cortical motor output during movement observation. Neuroreport.

[25] Montgomery K.J., Isenberg N., Haxby J.V. (2007). Communicative hand gestures and object-directed hand movements activated the mirror neuron system. Soc. Cogn. Affect. Neurosci..

[26] Muthukumaraswamy S.D., Johnson B.W., McNair N.A. (2004). Mu rhythm modulation during observation of an object-directed grasp. Cogn. Brain Res..

[27] Buccino G., Lui F., Canessa N., Patteri I., Lagravinese G., Benuzzi F., Porro C., Rizzolatti G. (2004). Neural circuits involved in the recognition of actions performed by nonconspecifics: An fMRI study. J. Cogn. Neurosci..

[28] Umilta M.A., Kohler E., Gallese V., Fogassi L., Fadiga L., Keysers C., Rizzolatti G. (2001). I know what you are doing: A neurophysiological study. Neuron.

[29] Den Ouden D.B., Fix S., Parrish T.B., Thompson C.K. (2009). Argument structure effects in action verb naming in static and dynamic conditions. J. Neurolinguist..

[30] Hauk O., Pulvermuller F. (2004). Neurophysiological distinction of action words in the fronto-central cortex. Hum. Brain Mapp..

[31] Indefrey P., Levelt W.J.M. (2004). The spatial and temporal signatures of word production components. Cognition.

[32] Chen X., Bin G., Daly I., Gao X. (2013). Event-related desynchronization (ERD) in the alpha band during hand mental rotation task. Neurosci. Lett..

[33] Hari R. (2006). Action–perception connection and the cortical mu rhythm. Prog. Brain Res..

[34] Jensen O., Goel P., Kopell N., Pohja M., Hari R., Ermentrout B. (2005). On the human sensorimotor-cortex beta rhythm: Sources and modeling. Neuroimage.

[35] McGarry L.M., Russo F.A., Schalles M.D., Pineda J.A. (2012). Audio-visual facilitation of the mu rhythm. Exp. Brain Res..

[36] Pineda J.A. (2005). The functional significance of my rhythms: Translating “seeing” and “hearing” into “doing”. Brain Res. Rev..

[37] Pineda J.A. (2008). Sensorimotor cortex as a critical component of an ‘extended’ mirror neuron system: Does it solve development, correspondence, and control problems in mirroring?. Behav. Brain Funct..

[38] Neuper C., Pfurtscheller G. (2001). Event-related dynamics of cortical rhythms: Frequency-specific features and functional correlates. Int. J. Psychophysiol..

[39] Pfurtscheller G., Lopes da Silva F.H. (1999). Event-related EEG/MEG synchronization and desynchronization: Basic principles. Clin. Neurophysiol..

[40] Onton J., Westerfield M., Townsend J., Makeig S. (2006). Imaging human EEG dynamics using independent component analysis. Neurosci. Biobehav. Rev..

[41] Jadhav P.N., Shanamugan D., Chourasia A., Ghole A.R., Acharyya A.A., Naik G. Automated detection and correction of eye blink and muscular artefacts in EEG signal for analysis of Autism Spectrum Disorder. Proceedings of the 2014 36th Annual International Conference of the IEEE on Engineering in Medicine and Biology Society (EMBC).

[42] Bhardwaj S., Jadhav P., Adapa B., Acharyya A., Naik G.R. Online and automated reliable system design to remove blink and muscle artefact in EEG. Proceedings of the 2015 37th Annual International Conference of the IEEE on Engineering in Medicine and Biology Society (EMBC).

[43] Mammone N., Morabito F.C. Independent component analysis and high-order statistics for automatic artifact rejection. Proceedings of the IEEE International Joint Conference on Neural Networks.

[44] Greco A., Mammone N., Morabito F.C., Versaci M. (2006). Kurtosis, Renyi’s entropy and independent component scalp maps for the automatic artifact rejection from EEG data. Int. J. Signal Process..

[45] Bowers A., Saltuklaroglu T., Harkrider A., Cuellar M. (2013). Suppression of the µ rhythm during speech and non-speech discrimination revealed by independent component analysis: Implications for sensorimotor integration in speech processing. PLoS ONE.

[46] Chuang C.H., Ko L.W., Jung T.P., Lin C.T. (2014). Kinesthesia in a sustained-attention driving task. Neuroimage.

[47] Makeig S., Delorme A., Westerfield M., Jung T.P., Townsend J., Courchesne E., Sejnowski T.J. (2004). Electroencephalographic brain dynamics following manually responded visual targets. PLoS Biol..

[48] Naeem M., Brunner C., Leeb R., Graimann B., Pfurtscheller G. (2006). Seperability of four-class motor imagery data using independent components analysis. J. Neural Eng..

[49] Tsai A.C., Jung T.P., Chien V.S.C., Savostyanov A.N., Makeig S. (2014). Cortical surface alignment in multi-subject spatiotemporal independent EEG source imaging. Neuroimage.

[50] Onton J., Makeig S. (2006). Information-based modeling of event-related brain dynamics. Prog. Brain Res..

[51] Makeig S., Debener S., Onton J., Delorme A. (2004). Mining event-related brain dynamics. Trends Cogn. Sci..

[52] Moreno I., De Vega M., León I. (2013). Understanding action language modulates oscillatory mu and beta rhythms in the same way as observing actions. Brain Cogn..

[53] Moreno I., De Vega M., León I., Bastiaansen M., Lewis A.G., Magyari L. (2015). Brain dynamics in the comprehension of action-related language. A time-frequency analysis of mu rhythms. Neuroimage.

[54] Vukovic N., Shtyrov Y. (2014). Cortical motor systems are involved in second-language comprehension: Evidence from rapid mu-rhythm desynchronisation. Neuroimage.

[55] Goodglass H., Kaplan E., Weintraub S., Segal O. (2001). Boston Naming Test.

[56] Nasreddine Z.S., Phillips N.A., Bedirian V., Charbonneau S., Whitehead V., Collin I., Cummings J.L., Chertkow H. (2005). The montreal cognitive assessment, MoCA: A brief screening tool for mild cognitive impairment. J. Am. Geriatr. Soc..

[57] Umla-Runge K., Zimmer H.D., Fu X., Wang L. (2012). An action video clip database rated for familiarity in China and Germany. Behav. Res. Methods.

[58] (2015). iMovie.

[59] Coltheart M. (1981). The MRC Psycholinguistic Database. Q. J. Exp. Psychol..

[60] Francis W.N., Kucera H. (1967). Computational Analysis of Present-Day American English.

[61] Brysbaert M., New B. (2009). Moving beyond Kucera and Francis: A critical evaluation of current word frequency norms and the introduction of a new and improved word frequency measure for American English. Behav. Res. Methods.

[62] Brysbaert M., Warriner A.B., Kuperman V. (2014). Concreteness ratings for 40 thousand generally known English word lemmas. Behav. Res. Methods.

[63] Psychology Software Tools, Inc. [E-Prime 2.0]. http://www.pstnet.com.

[64] Delorme A., Makeig S. (2004). EEGLAB: An open source toolbox for analysis of single-trial EEG dynamics including independent component analysis. J. Neurosci. Methods.

[65] Makeig S., Onton J., Luck S.J., Kappenman E.S. (2011). ERP features and EEG dynamics: An ICA perspective. The Oxford Handbook of Event-Related Potential Components.

[66] Manly J.J. (2008). Critical issues in cultural neuropsychology: Profit from diversity. Neuropsychol. Rev..

[67] Xie M., Singh K. (2013). Confidence distribution, the frequentist distribution estimator of a parameter: A review. Int. Stat. Rev..

[68] Benjamini Y., Hochberg Y. (1995). Controlling the false discovery rate: A practical and powerful approach to multiple testing. J. Royal Stat. Soc. Series B (Methodol.).

[69] Pulvermuller F. (2005). Brain mechanisms linking language and action. Nat. Rev. Neurosci..

[70] Rose M.L., Raymer A.M., Lanyon L.E., Attard M.C. (2013). A systematic review of gesture treatments for post-stroke aphasia. Aphasiology.

[71] Marangolo P., Caltagirone C. (2014). Options to enhance recovery from aphasia by means of non-invasive brain stimulation and action observation therapy. Exp. Rev. Neurother..

[72] Marangolo P., Cipollari S., Fiori V., Razzano C., Caltagirone C. (2010). Walking but not barking improves verb recovery: Implication for action observation treatment in aphasia rehabilitation. PLoS ONE.

[73] Marangolo P., Bonifazi S., Tomaiuolo F., Craighero L., Coccia M., Altoe G., Provinciali L., Cantagallo A. (2010). Improving language without words: First evidence from aphasia. Neuropsychologia.

[74] Bonifazi S., Tomaiuolo F., Altoe G., Ceravolo M.G., Provinciali L., Marangolo P. (2013). Action observation as a useful approach for enhancing recovery of verb production: New evidence from aphasia. Eur. J. Phys. Rehabil. Med..

[75] Faroqi-Shah Y., Graham L. (2011). Semantic treatment of verb naming in aphasia: Acquisition and generalization effects. Clin. Ling. Phon..

[76] Conroy P., Sage K., Lambon Ralph M.A. (2009). The effects of decreasing and increasing cue therapy on improving naming speed and accuracy for verbs and nouns in aphasia. Aphasiology.

[77] Fridriksson J., Morrow K.L., Moser D., Baylis G.C. (2006). Age-related variability in cortical activity during language processing. J. Speech Lang. Hear. Res..

